# Influential cited references in *FEMS Microbiology Letters*: lessons from Reference Publication Year Spectroscopy (RPYS)

**DOI:** 10.1093/femsle/fnz139

**Published:** 2019-06-24

**Authors:** Robin Haunschild, Johann Bauer, Lutz Bornmann

**Affiliations:** 1 Max Planck Institute for Solid State Research, Heisenbergstraße 1, D-70569 Stuttgart, Germany; 2 Max Planck Institute for Biochemistry, Am Klopferspitz 18, 82152 Martinsried, Germany; 3 Division for Science and Innovation Studies, Administrative Headquarters of the Max Planck Society, Hofgartenstr. 8, D-80539 Munich, Germany

**Keywords:** bibliometrics, cited references, reference publication year spectroscopy (RPYS), microbiology

## Abstract

The journal *FEMS Microbiology Letters* covers all aspects of microbiology including virology. On which scientific shoulders do the papers published in this journal stand? Which are the classic papers used by the authors? We aim to answer these questions in this study by applying the Reference Publication Year Spectroscopy (RPYS) analysis to all papers published in this journal between 1977 and 2017. In total, 16 837 publications with 410 586 cited references are analyzed. Mainly, the studies published in the journal *FEMS Microbiology Letters* draw knowledge from methods developed to quantify or characterize biochemical substances such as proteins, nucleic acids, lipids, or carbohydrates and from improvements of techniques suitable for studies of bacterial genetics. The techniques frequently used for studying the genetic of microorganisms in *FEMS Microbiology Letters*’ studies were developed using samples prepared from microorganisms. Methods required for the investigation of proteins, carbohydrates, or lipids were mostly transferred from other fields of life science to microbiology.

## Teaser

The aim of this study is to disclose the classic papers used by authors who published in the journal FEMS Microbiology Letters.

## INTRODUCTION

Citation analysis usually asks for the broad impact of publications—not only in the field in which the publications appeared but also in other fields. The broad impact measurement is reasonable, because one is interested in exceptional research that is useful for later research in many disciplines. However, research evaluation also asks for the relevance of publications in certain fields: for example, which papers were influential in the development of research in applied microbiology? On which shoulders is current research on bacterial genetics based on? To answer these and similar questions on the importance of publications and the origins in a field, the analysis of cited references (CRs)—as an alternative to the times-cited analysis—has been established (Bornmann and Marx 2013). The CR analysis focuses on the references cited in a given publication set (the backward view), whereas the times-cited analysis is based on the citation information given in bibliographic databases (the forward view) such as Scopus (Elsevier) or Web of Science (WoS, Clarivate Analytics).

The CR analysis should be applied by an expert in a given research field who is able to interpret the results sufficiently. The first step in the analysis is the selection of the publication set. Only an expert is able to collect reliably all publications of a certain research field. In the second step, the number of CRs which account for the reference publication years (RPYs) in a certain period (e.g. from 1900 until 1990) are plotted in a spectrogram. The peaks that become visible in the spectrogram are inspected in the third step of the analysis. The peaks point to publications (CRs) that have been cited in the initial publication set more frequently than other publications (CRs). An expert who is able to interpret the meaning of the publications (CRs) in the field inspects these publications and identifies the important publications in the historical context. This type of CR analysis which is mainly based on the interpretation of a spectrogram has been called reference publication year spectroscopy (RPYS, Marx *et al*. 2014).

In recent years, RPYS has been applied in various fields. Hou (2017) investigated the evolution process and historical roots of the topic ‘citation analysis’, Wray and Bornmann (2015) the origins of the philosophy of science, Gorry and Ragouet (2016) important publications in the research of Charles Dotter—the father of interventional radiology—and Bornmann, Haunschild and Leydesdorff (2018) the oeuvre of Eugene Garfield—the founder of the citation index. One of the RPYS studies using one of the largest data sets so far (more than 200 000 citing papers) examined the historical roots of climate change literature (Marx *et al*. 2017). RPYS has also been applied in the fields of dentistry and neurosciences (Yeung 2017; Yeung, Wong and Leung 2019). Very large publication sets can be analysed by sampling methods implemented in the CRExplorer (Haunschild *et al*. 2019). An overview of further studies based on RPYS can be found in Marx and Bornmann (2016).

Although several publications have appeared based on RPYS, the application of the method on the origin of the papers published in a single journal has scarcely been undertaken. One of such publications studies the historical roots of recent contributions to the journal *Ecological Economics* (Ballandonne 2018). Another one studies the CRs in three scientometric journals (Leydesdorff *et al*. 2014). We are not aware of any RPYS study regarding the field of microbiology. In this study, therefore, we investigate the origins of papers published in FEMS Microbiology Letters—a journal covering all aspects of microbiology, including virology (see https://academic.oup.com/femsle/pages/About, consulted on April 10, 2019). We wonder on which publications the researchers publishing in this journal frequently draw on.

## MATERIALS AND METHODS

### Materials

Our study is based on data from the Web of Science (WoS, Clarivate Analytics) web version and custom data of our in-house database derived from the Science Citation Index Expanded (SCI-E), Social Sciences Citation Index (SSCI), and Arts and Humanities Citation Index (AHCI) produced by Clarivate Analytics (Philadelphia, USA). Our in-house database contains the WoS publications since 1980. We exported all papers (without restriction to document type) published between 1980 and 2017 in *FEMS Microbiology Letters* from our in-house database and added the papers published in the same journal between 1977 and 1979 from the web-version of WoS. The papers were exported on July 10, 2018. In total, this data set contains 16 837 publications with 410 586 CRs. Times cited for the most influential CRs were collected from WoS on January 10, 2019.

### Software

Some years ago, the method RPYS was introduced to investigate the roots of research fields or topics (Marx *et al*. 2014). The software CRExplorer has been developed to facilitate these analyses; we used this programme to investigate the origins of papers from FEMS Microbiology Letters. This programme has been specifically developed for investigating CRs and to identify landmark papers (Thor *et al*.2016a,b). The programme is available at www.crexplorer.net (a comprehensive handbook explaining all functions is also available). With the programme Metaknowledge (McLevey and McIlroy-Young 2017), the Bibliometrix R package (Aria and Cuccurullo 2017), and the web tool RPYS i/o (Comins and Leydesdorff 2016), three other tools have been developed in recent years for doing CRs analyses, too. However, CRExplorer has a much broader functionality than the other tools. The most important function (for this study) is the disambiguation algorithm to cluster and merge equivalent CRs automatically. Furthermore, the possibility to use the CRExplorer script language to perform the analysis of the CRs (instead of the interactive interface) provides very clear documentation of the actions performed during the RPYS. The use of scripts increases the transparency of the statistical analysis and simplifies replications. The reader can download the WoS data and run the CRExplorer script in Fig. 2 to reproduce our results.

### Methodology

We used the CRExplorer with the script in Fig. 2 to import the WoS data into CRExplorer, cluster-equivalent CRs automatically with the Levenshtein threshold of 0.75 taking into account volume and page number, and merge them. We performed the RPYS analysis until the RPY 2015 to ensure a citation window of at least three years (we counted citations until the end of 2017). Finally, two sets of results are saved in different formats: CRE file, CR information file (CSV_CR) and CR data file (CSV_GRAPH)^1^ to plot the spectrogram using BibPlots (see: https://cran.r-project.org/web/packages/BibPlots/index.html) with each CR being referenced at least (i) five times and (ii) at least 50 times. We used the first export for plotting the spectrogram and the second one for identification of peak papers and papers which were referenced over many citing years rather frequently. A smaller NCR threshold value is chosen to sharpen the spectrogram and a larger NCR threshold is chosen for the advanced analysis to ensure that only fairly well-CRs need to be analysed. The CRE files can be opened interactively in the CRExplorer. The CR information file can be imported in a spreadsheet program to view the details of the CRs and to identify the peak papers. The CR data file with CRs referenced at least five times is useful to plot the spectrogram because noise of very lowly CRs is removed and the peaks are easier to identify than without removal of CRs. The removal of CRs referenced less than 50 times leads to a data set with 88 CRs which was inspected with the N_TOP10 indicator (which is explained in the next section below).

### Indicators used

We used two approaches to analyse the papers cited in *FEMS Microbiology Letters*: (i) We looked at the number of CRs (NCR) per RPY. This is the number of CRs accounting for a specific RPY. The five-year median deviation from the NCR in a specific RPY and the NCR curve were used to determine peak papers. (ii) It is a problem in RPYS that some publications (CRs) with considerable citation impact do not appear as peak papers. This is frequently the case with rather recent publications (CRs) which remain hidden behind many other recent publications (CRs). The CRExplorer provides advanced indicators for discovering them. We used the N_TOP10 indicator which shows the number of citing years in which a certain CR belongs to the 10% most frequently cited CRs. A detailed explanation of the advanced indicators in CRExplorer can be found in Thor *et al*. (2018) and in the CRExplorer handbook.

## RESULTS

**Figure 1.**
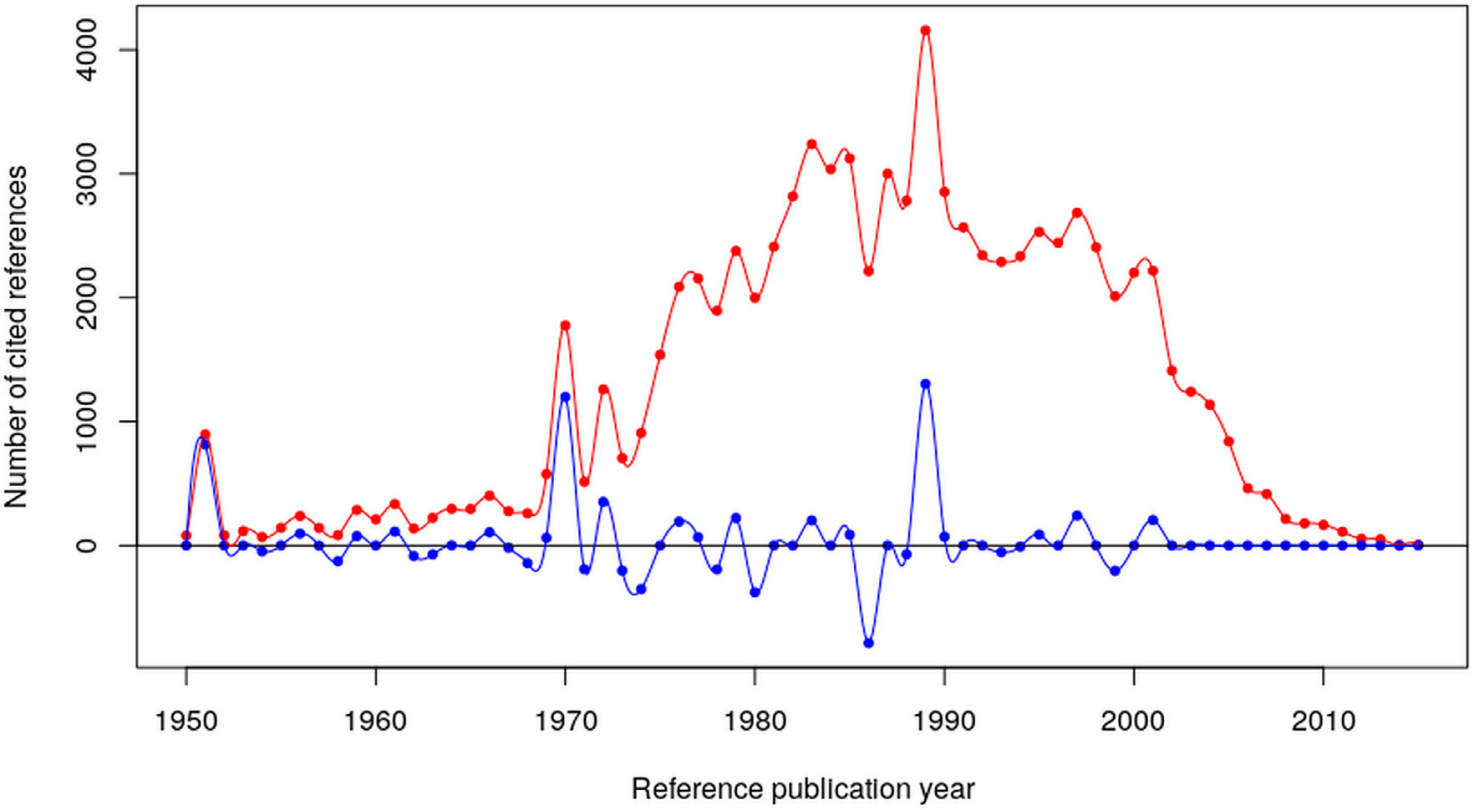
Annual distribution of cited references throughout the period 1950–2015 (based on cited references from papers published between 1977 and 2017 in *FEMS Microbiology Letters*). The red line shows the number of cited references and the blue line shows the five-year median deviation. The number of cited references curve is well below 100 before 1950. Therefore, the reference publication years before 1950 are not shown in Fig. 1.

The spectrogram of the RPYS analysis is shown in Fig. 1. The solid lines reveal the NCR in red and the five-year median deviations (RPY-2, RPY-1, RPY, RPY + 1, and RPY + 2) in blue. Both lines are used to identify the positions of the peaks in the spectrogram. We identified 15 peaks in the spectrogram of Fig. 1: in the RPYs 1951, 1956, 1959, 1961, 1966, 1970, 1972, 1976/1977, 1979, 1983, 1985, 1989, 1995, 1997, 2000/2001. In many cases, the peaks originate from publications (CRs) with much more impact than other publications (CRs) in the same RPY. These publications can be labeled as ‘peak publications’. The NCR curve is well below 100 before 1950. Therefore, the RPYs before 1950 are not shown in Fig. 1.

**Figure 2.**
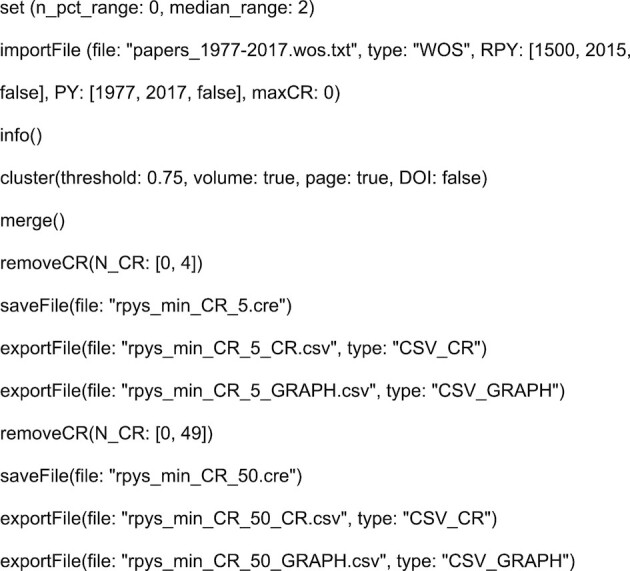
CRExplorer script to perform reference publication year spectroscopy on the cited references of papers published in *FEMS Microbiology Letters*.

The peak publications are listed in Table 1. The table does not only show the NCR for every publication, but also the total number of citations in WoS. We included those numbers additionally to draw a comparison between the impact the CRs had within all papers in the WoS and the papers published in *FEMS Microbiology Letters*. CR8, CR17, and CR22 are books. Of these, CR17 and CR22 are different editions (second and third) of the same book. Other editions appear also in other RPYs but not as peak papers. CR17 alone is already the most cited publication in *FEMS Microbiology Letters* (published between 1977 and 2017). This shows the importance of this book, which contains a number of molecular cloning techniques, for the papers published in *FEMS Microbiology Letters*. The rest of the CRs appeared in journals.

**Table 1.** Most frequently cited references (CRs) from peaks in Fig. 1, their identifier (#CR), reference publication year (RPY), cited reference with title, number of cited references (NCR), and total citations (TC) in Web of Science (WoS) ordered by RPY.

#CR	RPY	Cited reference with title	NCR	TC
CR1	1951	Lowry OH, 1951, *Journal of Biological Chemistry*, V193, P265	872	336 741
		Protein measurement with folin phenol reagent		
CR2	1956	Dubois M, 1956, *Analytical Chemistry*, V28, P350	100	32 767
		Colorimetric method for determination of sugars and related substances		
CR3	1959	Bligh EG, 1959, *Canadian Journal of Biochemistry and Physiology*, V37, P911	77	39 093
		A rapid method of total lipid extraction and purification		
CR4	1959	Miller GL, 1959, *Analytical Chemistry*, V31, P426	66	14 667
		Spectrophotometric determination of aldoses by an iodometric procedure		
CR5	1961	Marmur J, 1961, *Journal of Molecular Biology*, V3, P208	138	12 160
		Procedure for isolation of deoxyribonucleic acid from micro-organisms		
CR6	1966	Stanier RY, 1966, *Journal of General Microbiology*, V43, P159	36	2512
		Aerobic pseudomonads—a taxonomic study		
CR7	1970	Laemmli UK, 1970, *Nature*, V227, P680	1270	245 097
		Cleavage of structural proteins during the assembly of the head of bacteriophage T4		
CR8	1972	Miller JH, 1972, *Experiments in molecular genetics*. Cold Spring Harbor, N.Y.: Cold Spring Harbor Laboratory	443	> 27 000**
CR9	1976	Bradford MM, 1976, *Analytical Biochemistry*, V72, P248	802	201 681
		A rapid and sensitive method for the quantitation of microgram quantities of protein utilizing the principle of protein-dye binding		
CR10	1977	Sanger F, 1977, *Proceedings of the National Academy of Sciences of the United States of America* (PNAS), V74, P5463	432	67 222
		DNA Sequencing with Chain-Terminating Inhibitors		
CR11	1979	Towbin H, 1979, *Proceedings of the National Academy of Sciences of the United States of America* (PNAS), V76, P4350	274	55 412
		Electrophoretic transfer of proteins from polyacrylamide gels to nitrocellulose sheets: procedure and some applications		
CR12	1979	Birnboim HC, 1979, *Nucleic Acids Research*, V7, P1513	270	13 103
		Rapid alkaline extraction procedure for screening recombinant plasmid DNA		
CR13	1983	Simon R, 1983, *Bio-Technology*, V1, P784	220	5373
		A broad host range mobilization system for invivo genetic-engineering—transposon mutagenesis in gram-negative bacteria		
CR14	1983	Hanahan D, 1983, *Journal of Molecular Biology*, V166, P557	208	8970
		Studies on transformation of Escherichia coli with plasmids		
CR15	1985	Yanischperron C, 1985, *Gene*, V33, P103	283	14 649
		Improved m13 phage cloning vectors and host strains—nucleotide-sequences of the m13mp18 and puc19 vectors		
CR16	1985	Hopwood DA, 1985, *Genetic manipulation of Streptomyces: a laboratory manual*. The John Innes Foundation, Norwich, UK, and Cold Spring Harbour Laboratory	144	2650
CR17	1989	Sambrook J, 1989, *Molecular cloning: a laboratory manual* (2. edition). Cold Spring Harbor, N.Y.: Cold Spring Harbor Laboratory	1687*	> 140 000**
CR18	1995	Kovach ME, 1995, *Gene*, V166, P175	78	2097
		4 new derivatives of the broad-host-range cloning vector pBBR1MCS, carrying different antibiotic-resistance cassettes.		
CR19	1995	Amann RI, 1995, *Microbiological Reviews*, V59, P143	68	5965
		Phylogenetic identification and in situ detection of individual microbial cells without cultivation		
CR20	1997	Altschul SF, 1997, *Nucleic Acids Research*, V25, P3389	346	47 096
		Gapped BLAST and PSI-BLAST: a new generation of protein database search programs.		
CR21	2000	Datsenko KA, 2000, *Proceedings of the National Academy of Sciences of the United States of America* (PNAS), V97, P6640	139	8239
		One-step inactivation of chromosomal genes in Escherichia coli K-12 using PCR products.		
CR22	2001	Sambrook J, 2001, *Molecular cloning: a laboratory manual* (3. edition). Cold Spring Harbor, N.Y.: Cold Spring Harbor Laboratory	203	> 27 000**
CR23	2001	Livak KJ, 2001, *Methods*, V25, P402	70	67,202
		Analysis of relative gene expression data using real-time quantitative PCR and the 2(-Delta Delta C(T)) Method		

* Two variants of the same CR with NCR = 1330 and NCR = 357.

** CR8, CR17 and CR22 are not source papers of the WoS. The TC number had to be estimated via the cited references search.

Only two of the 20 CRs in Table 1 (CR6 and CR14) unveil microbiological phenomena. While in CR6 the classification of pseudomonads is explained, CR14 contains observation on *Escherichia coli* transformation by various plasmids under different conditions. Eighteen of the 20 CRs in the table describe certain methods. The publications indicated as CR1-5, CR7-13, and CR15-23 contain information about newly developed methods. They can be divided in three groups: The first group comprises papers describing methods, which are very useful for quantification (CR1 and CR9) and characterization of proteins (CR7 and CR11). Of those, only the authors of CR7 and CR11 studied microbiological topics and used samples prepared from microorganisms to develop their methods. However, all the techniques described in CR1, CR7, CR9, and CR11 were applied within the community studying proteins in any way. Three of these papers (CR1, CR7, and CR9) were cited more than 200 000 times in WoS each (TC in Table 1). Between 0.25% and 0.5% of the citations (TC) are accounted to *FEMS Microbiology Letters*. Considering the high number of journals publishing manuscripts about proteins, it appears that the influence of these cited papers on publications published in *FEMS Microbiology Letters* is above average.

Other papers cited very frequently in *FEMS Microbiology Letters* describe the development and first application of techniques, which are useful in investigation of sugars (CR2 and CR4) and lipids (CR3). Although these techniques were developed outside of microbiology, they are important in microbiology as in other research fields studying carbohydrates or lipids. A number of peak papers in *FEMS Microbiology Letters* describe techniques, which are useful for studying the genetics of bacteria. All these techniques were developed inside microbiology using different microbes or phages and their DNA. Early papers in Table 1 describe methods of DNA isolation and DNA sequencing (CR5 and CR10). Later papers deal with improvements of DNA cloning (CR15 and CR18) and DNA transfer (CR12, CR13, and CR14). Also in CR20 and CR23, methods are described that are applicable in bacterial genetics. These methods are essential for the interpretation of DNA sequence analysis. CR21 describes a new way to determine types of bacteria. This was the starting point for determining species of bacteria without interims culturing. Finding the right culturing conditions for a mixture of bacteria present, e.g., in biofilms is a gamble. Thus, the identification of a species of bacteria as well as early recognition of pathogens became more reliable and—obviously—was often applied in studies published in *FEMS Microbiology Letters*.

As outlined in Section 2.4, we additionally used advanced indicators provided in CRExplorer to identify landmark publications used by papers published in *FEMS Microbiology Letters*. We are interested in those publications which were very useful over several citing years (and belong to the top-cited publications). CRs 8, 10–15, 17, and 20 from Table   1 were very useful in more than five citing years. Nine additional CRs have been identified by CRExplorer as landmark publications which were highly cited in more than five citing years. These additional CRs are listed in Table 2: one book and eight journal papers. Only one of these publications (CR32) describes a new system of classification of blue-green algae, which was of interest for the studies published in *FEMS Microbiology Letters* afterwards. The others explain methods which are helpful in microbiology: six are related to genetics of microorganisms (CR25, CR26, CR27, CR31) or bioinformatics (CR24, CR28). Two CRs describe techniques which are useful in lipid chemistry (CR30) or cultivation of bacteria (CR29).

**Table 2.** Cited references (CRs) which belong in more than five citing years to the 10% most frequently cited publications (and are not listed in Table 1), their identifier (#CR), reference publication year (RPY), cited reference with title, number of cited references (NCR), and N_TOP10 value ordered by N_TOP10.

#CR	RPY	Cited reference with title	NCR	N_TOP10
CR24	1987	Saitou N, 1987, *Molecular Biology and Evolution*, V4, P406	212	12
		The neighbor-joining method—a new method for reconstructing phylogenetic trees		
CR25	1975	Southern EM, 1975, *Journal of Molecular Biology*, V98, P503	197	8
		Detection of specific sequences among DNA fragments separated by gel-electrophoresis		
CR26	1974	Beringer JE, 1974, *Journal of General Microbiology*, V84, P188	69	7
		R factor transfer in rhizobium-leguminosarum		
CR27	1982	Maniatis T, 1982, *Molecular cloning: a laboratory manual*. CSHL Press	410	7
CR28	1990	Altschul SF, 1990, *Journal of Molecular Biology*, V215, P403	316	7
		Basic local alignment search tool		
CR29	1960	De Man J. C., 1960, *Jour Appl Bact*, V23, P130	71	7
		A medium for the cultivation of lactobacilli		
CR30	1965	Westphal O., 1965, *Method Carbohyd Chem*, V5, P83	60	7
		Bacterial lipopolysaccharides extraction with phenol–water and further applications of the procedure		
CR31	1963	Saito H, 1963, *Biochimica et Biophysica Acta*, V72, P619	52	7
		Preparation of transforming deoxyribonucleic acid by phenol treatment		
CR32	1971	Stanier RY, 1971, *Bacteriological Reviews*, V35, P171	54	6
		Purification and properties of unicellular blue–green algae (order Chroococcales)		

## DISCUSSION

In this study, we investigated the most influential CRs of the journal *FEMS Microbiology Letters*. It is one of the few studies in scientometrics which target the most influential CRs of a journal (in most of the cases small fields or topics have been investigated). Furthermore, we are not aware of another RPYS study in the field of microbiology. Taken together, our results indicate that methods developed to quantify or characterise biochemical substances such as proteins, nucleic acids, lipids, or carbohydrates and improvements of techniques suitable for studies of bacterial genetics were important for the studies published in *FEMS Microbiology Letters*. The techniques frequently used for studying the genetic of microorganisms in *FEMS Microbiology Letters*’ studies were developed using samples prepared from microorganisms. Methods required for the investigation of proteins, carbohydrates or lipids were mostly transferred from other fields of life science to microbiology.

The question remains whether RPYS is reliably able to identify the roots of research fields: do experts in the fields come to similar conclusions? Marx *et al*. (2014) used research on Graphene to undertake RPYS. They compared the results of the RPYS with the results of a literature overview on Graphene research pointing to important historical papers. The comparison showed that the expert view discovered similar historical roots as the RPYS. A similar result has been presented by Comins and Leydesdorff (2017). They assessed whether the results of RPYS ‘converge with expert opinions on research milestones driving biomedical innovation in the treatment of Basal Cell Carcinoma. Our results show that these algorithms successfully identify the majority of milestone papers detailed by experts … thereby validating the power of these algorithms to converge on independent opinions of seminal scientific works derived by subject matter experts’ (p. 1495).

Future work could study the influential CRs of other journals or complete journal sets, as those used by Clarivate Analytics to develop a field-classification scheme for the WoS covering a broad spectrum of scientific fields. The first approach is especially interesting if journals may represent the research in a specific field. Journal sets representing complete fields can be used to enlarge the journal approach applied in this study and to identify the roots of broader fields. Recently, Thor *et al*. (2019) studied the highly influential publications of all WoS subject categories, i.e., journal sets. The results can be found online at www.crexplorer.net (WoS Landmark Papers).

## Supplementary Material

fnz139_Supplemental_File

## Footnotes

1 The CSV files can be downloaded at: https://tinyurl.com/yaqugfma
